# Pharmacologic inhibition of mTORC1 mimics dietary protein restriction in a mouse model of lactation

**DOI:** 10.1186/s40104-020-00470-1

**Published:** 2020-06-29

**Authors:** Virginia L. Pszczolkowski, Steven J. Halderson, Emma J. Meyer, Amy Lin, Sebastian I. Arriola Apelo

**Affiliations:** 1 Department of Animal and Dairy Sciences, University of Wisconsin-Madison, Madison, WI USA; 2 Endocrinology and Reproductive Physiology Graduate Training Program, University of Wisconsin-Madison, Madison, WI USA

**Keywords:** Amino acids, Lactation, Mammary, Mouse model, mTORC1, Rapamycin

## Abstract

**Background:**

Understanding the mechanisms of N utilization for lactation can lead to improved requirement estimates and increased efficiency, which modern dairy diets currently fail to maximize. The mechanistic target of rapamycin complex 1 (mTORC1) is a central hub of translation regulation, processing extra- and intra-cellular signals of nutrient availability and physiological state, such as amino acids and energy. We hypothesized that dietary amino acids regulate lactation through mTORC1, such that inhibition of mTORC1 will lead to decreased lactation performance when amino acids are not limiting. Our objectives were to assess lactation performance in lactating mice undergoing dietary and pharmacologic interventions designed to alter mTORC1 activity.

**Methods:**

First lactation mice (*N* = 18; *n* = 6/treatment) were fed an adequate protein diet (18% crude protein), or an isocaloric protein-restricted diet (9% crude protein) from the day after parturition until lactation day 13. A third group of mice was fed an adequate protein diet and treated with the mTORC1 inhibitor rapamycin (4 mg/kg every other day) intraperitoneally, with the first two groups treated with vehicle as control. Dams and pups were weighed daily, and feed intake was recorded every other day. Milk production was measured every other day beginning on lactation day 4 by the weigh-suckle-weigh method. Tissues were collected after fasting and refeeding.

**Results:**

Milk production and pup weight were similarly decreased by both protein restriction and rapamycin treatment, with final production at 50% of control (*P* = 0.008) and final pup weight at 85% of control (*P* < 0.001). Mammary phosphorylation of mTORC1’s downstream targets were decreased by protein restriction and rapamycin treatment (*P* < 0.05), while very little effect was observed in the liver of rapamycin treated mice, and none by protein restriction.

**Conclusions:**

Overall, sufficient supply of dietary amino acids was unable to maintain lactation performance status in mice with pharmacologically reduced mammary mTORC1 activity, as evidenced by diminished pup growth and milk production, supporting the concept that mTORC1 activation rather than substrate supply is the primary route by which amino acids regulate synthesis of milk components.

## Introduction

Dairy cows and other ruminant livestock are incredible converters of low-quality feeds [1] into milk, meat, and fiber, and when properly managed have an important role in sustainable food systems [2]. However, in modern high-production settings, dairy cattle are frequently fed above their nitrogen requirements in an attempt to maximize production at the expense of nutrient efficiency [3]. According to a meta-analysis by Hristov et al. [4], on average only 25% of all feed nitrogen (N) is sequestered as milk protein, with the majority of the remaining N excreted into the environment after post-absorptive losses.

As ruminants, dairy cattle pose a more complicated nutritional system than do monogastrics, as rumen protein degradability, microbial metabolism, and N recycling to the rumen come into play. However, it is post-absorption where lactating dairy cattle experience the largest N losses, falling behind monogastrics in N efficiency [5]. Current understanding of AA use by the mammary glands is largely centered around the role that AA play as substrate (i.e., as building blocks) on milk protein synthesis [6]. However, mounting evidence suggests that supply of AA to the mammary glands is not the most important or physiologically relevant factor in governing milk protein synthesis, but rather the glands respond to a host of stimuli to determine demand for individual AA. Across species, mammary glands’ extraction efficiency is always far below 100% for all AA, even in circumstances in which canonically limiting amino acids are restricted, as has been shown with Lys in lactating sows [7] and Met in lactating goats [8]. More importantly, the mammary glands exhibit plasticity in response to changes in AA supply: for example, when His was restricted in lactating goats, mammary clearance of His increased by 43-fold while clearance of other AA was concomitantly decreased [9]. Clearance rates of AA by the mammary glands respond to changes in other nutrients as well: both Rius et al. [10] and Omphalius et al. [11] reported increases in AA clearance by the mammary glands in response, respectively, to abomasal starch infusion and increased dietary energy, without increasing AA supply.

Before their fates can be sealed in a casein micelle or otherwise metabolized, specific AA act as signaling molecules for transduction pathways that orchestrate the demand for milk synthesis based on the systemic metabolic state of the animal [12–14]. Of these pathways, the mechanistic target of rapamycin complex 1 (mTORC1) pathway plays a central role, integrating information from cellular stressors, growth factors, and nutrients for downstream regulation of anabolic and catabolic processes such as protein and fat synthesis and autophagy [15]. The serine/threonine protein kinase mTOR forms two protein complexes: mTORC1, uniquely composed of Raptor and PRAS40; and mTORC2, containing Rictor, mSin1, and Protor-1/2. Shared between the two complexes are mTOR itself, mLST8, and Deptor. The antibiotic/antifungal rapamycin, also known as sirolimus, for which the protein kinase is named, inhibits mTORC1 by complexing with FKBP12 to disrupt the raptor-mTOR interaction [16]. While mTORC2 is insensitive to acute rapamycin treatment due to the absence of raptor [17], upon chronic treatment rapamycin indirectly inhibits mTORC2 structure and function [18]. This off-target effect of rapamycin can be mitigated by intermittent treatment with the drug, allowing for longer-term use in both research and medical settings [19].

In both *in vitro* and *in vivo* models of the lactating mammary gland, mTORC1 activity in response to individual AA has been shown to correlate with the rate of casein synthesis [20–22]. Recently, the mechanistic reasons for this correlation have begun to be elucidated *in vitro* [23–25]. As well, beyond contributing to milk protein synthesis, *in vitro* stimulation of mTORC1 by AA has shown to regulate milk fat synthesis [13, 26]. However, *in vivo* evidence for whether or not mTORC1 plays a causal role in the regulation of lactation by AA is still lacking.

For increasing understanding of the fundamental mechanisms that can ultimately lead to development of targeted nutritional or pharmacological interventions in dairy cattle, a murine model of lactation has the advantage over bovine of increased economic efficiency, environmental consistency, sample size, and speed. Tissue-specific genetic manipulations and chemically-defined dietary alterations are standard fare in mouse research, allowing for tight control of experimental conditions. Lactation studies employing such models have previously yielded translatable results [22, 27, 28] that offer a direct path to further research in dairy cows [29, 30] and other species. Mice, as monogastric and litter-bearing animals, do have their limitations in translating results to cattle, but foundational research with this model gives us the ability to rapidly gain insight into the post-absorptive and molecular mechanisms that govern lactation across species.

We hypothesized that dietary amino acids regulate lactation through mTORC1, such that inhibition of mTORC1 will lead to decreased lactation performance when amino acids are not limiting. To test this hypothesis, our objectives were to assess lactation performance in mice undergoing dietary and pharmacologic interventions designed to alter mTORC1 signaling.

## Materials & methods

### Animals and experimental design

At parturition (lactation day zero, LD0), first lactation CL57B6/J mouse dams were randomly assigned (*N* = 18; *n* = 6/treatment) to one of three treatments: AP (adequate protein diet, 18% crude protein (CP) from casein with adequate energy for lactation, Envigo TD171019; Table 1); PR (protein restricted diet, 9% CP from casein with adequate energy for lactation, Envigo TD171020; Table 1); AP-R (AP diet, plus dams treated with the mTORC1 inhibitor rapamycin in ethanol at 4 mg/kg every other day (EOD) starting LD2 via intraperitoneal (IP) injection). AP and PR groups received vehicle only (5% PEG 400, 5% Tween 20, 0.9% NaCl in sterile water and AP-R equivalent volume ethanol) on the same injection schedule as AP-R. Dams had been bred to CL57B6/J males.

**Table 1:** Experimental diets components and macronutrients

Component, g/kg			AP^a^	PR^a^
Casein			207.0	103.5
Corn starch			386.5	489.1
Maltodextrin			132.0	132.0
Sucrose			100.0	100.0
Soybean meal			70.0	70.0
Cellulose			50.0	50.0
Mineral mix^b^			18.36	18.36
Calcium phosphate, dibasic			9.87	13.07
Calcium carbonate			8.38	6.05
Ferric citrate			0.3	0.3
Vitamin mix^c^			15.0	15.0
Choline bitartrate			2.5	2.5
TBHQ			0.014	0.014
Food color			0.1	0.1
kcal/g			3.7	3.7
	AP		PR	
Macronutrient, %	by weight	kcal from	by weight	kcal from
Protein	18.0	19.4	9.0	9.7
Carbohydrate	58.8	63.2	68.0	73.1
Fat	7.2	17.4	7.1	17.2

^a^AP = 18% crude protein diet, PR = 9% crude protein diet

^b^Envigo Teklad Diets, Madison, WI, catolog no. 98057

^c^Envigo Teklad Diets, Madison, WI, catolog no. 94047

Litters were standardized to 5 pups on LD1. Any pup mortality beyond this standardization was recorded and included in the analysis. Diets were applied on LD2 and food consumption was measured EOD. Dam weights and litter weights were taken every day starting on LD2. Litter weights were standardized to number of pups by dividing by the number of pups on each day of lactation. Starting on LD5, milk production was measured every other day by the weigh-suckle-weigh method [31]. Briefly, pups were separated from dams by placement in a ventilated pipette tip box within their home cage starting at 07:30 for 4 h, after which whole litters were weighed, returned to dams, and allowed to suckle for 45 min. Litters were then weighed again to estimate milk production during this single suckling period.

### Sample collection and preparation

On LD13, dams were fasted for 4 h starting at 07:00. At this time, pups were separated following the weigh-suckle-weigh protocol. After the 45 min suckling period, pups were again separated, and dams were refed for 4 h before being euthanized by cervical dislocation following terminal maxillary vein bleeding [32]. This system of fasting, suckling, and feeding was utilized to ensure all dams were in the fed state and to keep variation in mammary glands activity minimized at the time of tissue collection. The fourth mammary glands pair and left lateral lobe of the liver from each dam was collected and flash-frozen in liquid nitrogen within 3 min of euthanasia, and stored at − 80 °C until analysis. Pups were euthanized by decapitation and 1 liver/litter was randomly collected, flash-frozen in liquid nitrogen, and stored at − 80 °C for liver rapamycin analysis.

AP-R pup and dam liver samples were shipped overnight in dry ice to the Nathan Shock Center – Analytical Pharmacology Core Lab (UT Health, San Antonia, TX, USA) for rapamycin analysis.

Approximately equally sized samples of frozen mammary gland and liver tissues, prepared on dry ice, were lysed in 1 mL RIPA lysis buffer (50 mmol/L HEPES, 40 mmol/L NaCl, 2 mmol/L EDTA, 1.5 mmol/L sodium orthovanadate, 50 mmol/L NaF, 10 mmol/L sodium pyrophosphate, 10 mmol/L sodium 2-glycerophosphate at pH 7.4) using a Mini-Beadbeater-24 (BioSpec Products, Inc., Bartlesville, OK). Lysed samples were centrifuged at 4 °C and 18,000 RCF for 10 min to remove tissue debris, then repeatedly passed to new tubes and centrifuged at 0 °C and 12,000 RCF for 15 min to defat. Defatted protein samples were analyzed for total protein content by BCA Protein Assay (Thermo Fisher Scientific, Waltham, MA,  USA) following manufacturer instructions. Subsamples of these lysates were respectively standardized to 1.5 mg/mL with 5× sample buffer for gel electrophoresis [33], or 2.0 mg/mL with RIPA lysis buffer for further processing.

### Western blotting

Denatured protein from tissue (30 μg) were electrophoretically separated in 8% and 16% Novex Tris-glycine mini gels (Thermo Fisher Scientific, Waltham, MA, USA) and wet-transferred onto nitrocellulose membranes. Membranes were blocked with Odyssey blocking buffer (LI-COR Biosciences, Lincoln, NE, USA) diluted 1:1 with TBS for 1 h, then incubated overnight at 4 °C with primary antibodies for the total and phosphorylated forms of protein kinase B (Akt, Ser473), ribosomal protein S6 kinase beta-1rp (S6K1,Thr389), eukaryotic translation initiation factor 4E-binding protein 1 (4E-BP1, Ser65), and ribosomal protein S6 (rpS6, Ser240/244) (total catalog #s 2920, 2708, 9644, 2317; and phosphorylated catalog #s 4060, 9234, 9451, 5364, respectively, Cell Signaling Technology, Danvers, MA, USA) and for β-tubulin (catalog # 86298) in Odyssey blocking buffer diluted 1:1 with TBST. Of note, 4E-BP1 (Ser65) corresponds to the numbering of the human isoform; in mice this phosphosite is at Ser64.

Primary antibody-bound membranes were incubated with relevant fluorescent (goat anti-mouse IgG IRDye 680 RD #68070, goat anti-rabbit IgG IRDye 800CW #32211, LI-COR Biosciences) or chemiluminescent (HRP-linked goat anti-rabbit IgG #7074, HRP-linked goat anti-mouse #7076, Cell Signaling Technology) secondary antibodies for 1 h at room temperature before imaging on an Odyssey Fc imaging system (LI-COR Biosciences). Band intensities were quantified with ImageStudio software (LI-COR Biosciences). Phosphorylation of target proteins was calculated as the phosphorylated:total ratio of each target.

### Amino acid analysis

Plasma samples (30 μg) and lysed tissue samples (2.0 mg/mL protein, 24 μg) were combined with an internal standard algal 13C amino acid mix (6 μg; Cambridge Isotope Laboratories, Inc.; catalog no. CLM-1548-PK, CLM-8699-H-PK, CLM-4290-H-PK, CLM-1822-H-PK) before deproteinization with 1 mol/L perchloric acid (final concentration of 0.5 mol/L). Samples were prepared for analysis and separated using the EZ:faast kit (Phenomenex, catalog no. KH0–7337) and the Nexera-i LC-2040C (Shimadzu, Kyoto, Japan). Mobile phases were 10 mmol/L ammonium formate in water (A) and 10 mmol/L ammonium formate in methanol (B), with a 68% B gradient for 0–13 min, 83% B gradient for 13–13.01 min, and 68% B gradient for 13.02–17 min. Single quadrupole, electrospray ionization mass spectrometry was conducted with the LCMS-2020 (Shimadzu, Kyoto, Japan).

### Statistical analysis

All data analysis was performed in RStudio (version 1.1.414). Live data were analyzed by ANOVA with repeated measures with the *gls* function in the emmeans package [34]. Phosphorylation and AA data were analyzed by ANOVA followed by post-hoc Dunnett comparisons against AP with the multcomp package [35]. Rapamycin content of dam and pup livers were compared via student’s *t* test. Significance was set at *P* ≤ 0.05, and tendencies at 0.05 ≤ *P* ≤ 0.1.

## Results

### Live animal data

**Fig. 1:**
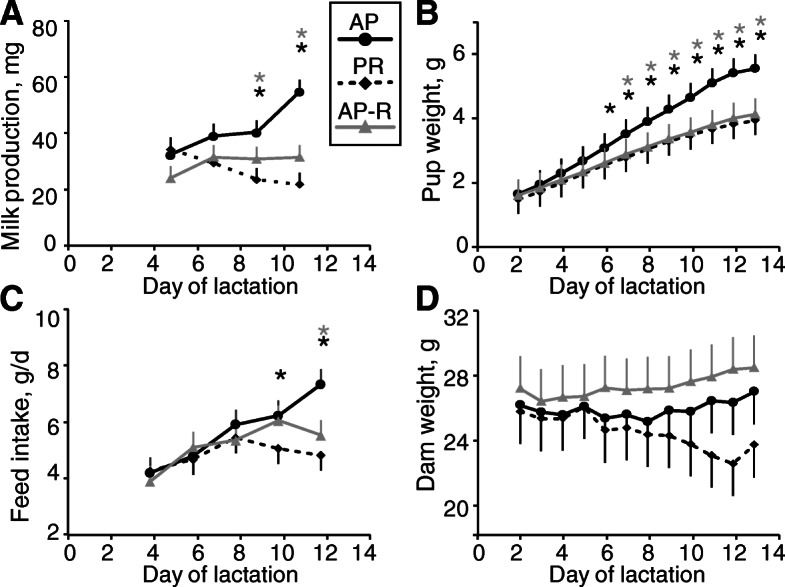
Rapamycin treatment mimics dietary protein restriction on lactation performance. **a** Milk production normalized to milk intake per pup during 1 bout of nursing. **b** Litter weight by pup. **c** Dam feed intake. **d** Dam weight. Data are means ± pooled SEM. Corresponding asterisks indicate *P* < 0.05 against AP control by two-way ANOVA with repeated measures

Milk production for both PR and AP-R by the weigh-suckle-weigh method began to decrease relative to AP starting at LD9, and by LD11 (*P* = 0.008) was approximately 50% of AP (Fig. 1a). Coupled to this lower peak milk production capacity for both PR and AP-R was an identical 15% decrease in pup weight by LD13 (*P* < 0.001), as compared to AP (Fig. 1b).

Dam food consumption decreased for both PR and AP-R relative to AP which consistently climbed until LD13, with PR falling below AP by LD10 (*P* = 0.04) and AP-R by LD12 (*P* = 0.002, Fig. 1c). Dam weight did not significantly change for any treatment throughout lactation, but AP-R maintained a consistently numerically higher weight than AP as lactation progressed, while PR resulted in a numerically continuously decreasing dam weight starting around LD6 (Fig. 1d).

### Mammary gland and liver mTORC1 signaling

**Fig. 2:**
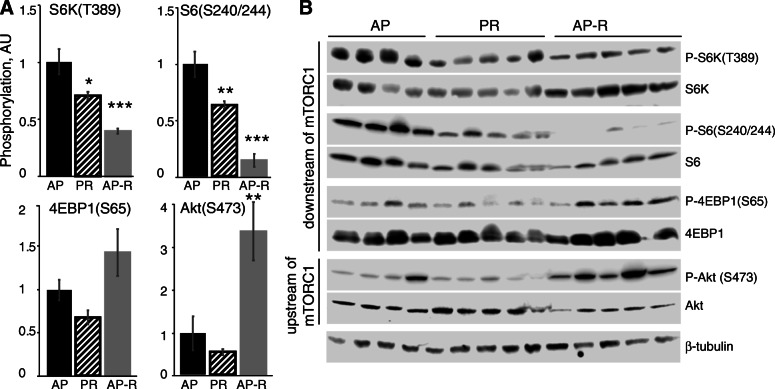
Dietary protein and rapamycin differentially affect mTORC1 signaling in the lactating mammary glands. **a** Western blot scans of LD13 dam mammary glands for phosphorylated and total forms of mTORC1 pathway effectors. **b** Relative phosphorylation of proteins as phosphorylated:total ratio. Data are mean ± SEM. * 0.05 < *P* < 0.01; ** 0.01 < *P* < 0.001; *** *P* < 0.001 against AP control by one-way ANOVA with Dunnet *post hoc*

Phosphorylation of the mTORC1 substrate S6K1(Thr389) in the mammary glands (Fig. 2) was reduced from AP by PR (30%, *P* = 0.01) and AP-R (61%, *P* < 0.001). Similarly, rpS6(S240/244) phosphorylation was reduced by PR (37%, *P* = 0.007) and AP-R (85%, *P* < 0.001). Surprisingly, phosphorylation of 4EBP(Ser65) was only numerically reduced by PR, and numerically increased by AP-R. Akt(Ser473) phosphorylation status was not altered from AP levels by PR, but AP-R resulted in a 350% increase in phosphorylation (*P* = 0.009).

**Fig. 3:**
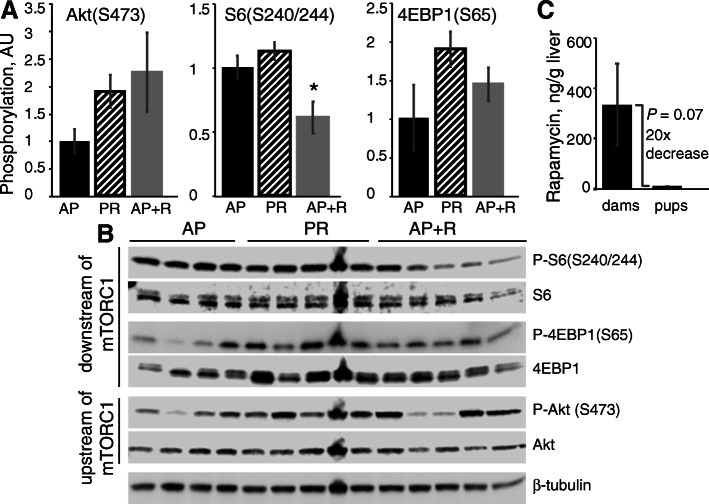
Dietary protein and rapamycin have limited effects on liver mTORC1 signaling in lactating mice. **a** Relative phosphorylation of proteins as phosphorylated:total ratio. **b** Western blot scans of LD13 dam livers for phosphorylated and total forms of mTORC1 pathway effectors. Data are mean ± SEM. * 0.05 < *P* < 0.01 against AP control by one-way ANOVA with Dunnet *post hoc*

In the liver (Fig. 3), only phosphorylation of rpS6(Ser240/244) was affected, with AP-R resulting in a 40% decrease in phosphorylation relative to AP (*P* = 0.04). No significant effects of PR or AP-R were seen in phosphorylation of either Akt(Ser473) or 4EBP(Ser65); S6K(Thr398) was not detectable with the antibody used.

### Pup exposure to rapamycin

While AP and PR dams and pups had no detectable rapamycin in their livers (data not shown), AP-R dams and pups livers both contained rapamycin, with dams averaging 333 ± 163 ng/g and pups averaging 11.0 ± 1.00 ng/g (Fig. 3c). Due to high variation within dam liver rapamycin content, dams and pups liver rapamycin content only tended (*P* = 0.07) to be different.

### Amino acid concentrations

Refed plasma amino acid levels were largely consistent across treatment groups, with no differences between any essential AA (EAA). Of the nonessential AA (NEAA), Ala, Asp, and Gln tended 0.05 <  *P* < 0.01 to be greater for AP + R relative to AP, with relative differences of 378, 2.55, and 220 μmol/L respectively (Table 2).

**Table 2:** Free AA concentrations in refed dam plasma on LD13^1,2^

Amino acid	AP	PR		AP-R		
	μmol/L	μmol/L	*P-*value^3^	μmol/L	*P-*value^3^	SEM
EAA						
Arginine	108	124	0.75	91	0.61	23.0
Histidine	105	117	0.73	116	0.79	19.1
Isoleucine	131	96	0.53	200	0.14	37.0
Leucine	265	57	0.19	70	0.22	114
Lysine	786	701	0.56	881	0.50	94.3
Methionine	96	59	0.51	132	0.51	37.0
Phenylalanine	89	71	0.27	82	0.79	12.0
Threonine	260	243	0.93	362	0.19	61.0
Tryptophan	64	50	0.75	76	0.78	22.0
Valine	224	141	0.21	325	0.11	51.2
NEAA						
Alanine	631	974	0.12	1001	0.08	174
Aspartate	3.7	4.0	0.95	6.3	0.08	1.17
Glutamate	25	14	0.20	35.5	0.24	6.79
Glutamine	661	762	0.45	882	0.06	91.9
Proline	166	154	0.94	202	0.64	54.5
Serine	181	244	0.28	215	0.65	44.4
Tyrosine	292	185	0.31	387	0.38	78.7

^1^AP = 18% crude protein diet, PR = 9% crude protein diet, AP-R = 18% crude protein diet coupled with rapamycin injections

^2^Data are means and pooled SEM, *n* = 6/treatment

^3^*P-*values correspond to difference between respective treatment and AP by ANOVA with Dunnett *post hoc*

The mammary tissue exhibited similarly consistent amino acid levels across treatment groups, but with high inter-animal variation (Table 3). Only Ala and Gln showed differences, with both increased (*P* = 0.05) relative to AP by AP-R, respectively by 20.2 and 7.64 μmol/g protein. Gln also tended to increase for PR relative to AP (*P* = 0.08).

**Table 3:** Free AA concentrations in refed dam mammary tissue on LD13^1,2^

Amino acid	AP	PR		AP-R		
	μmol/g protein	μmol/g protein	*P-*value^3^	μmol/g protein	*P-*value^3^	SEM
EAA						
Arginine	1.98	3.57	0.61	1.88	0.98	1.91
Histidine	7.13	5.18	0.83	2.12	0.73	4.04
Isoleucine	4.31	4.84	0.82	4.50	0.97	1.05
Leucine	3.87	22.0	0.73	20.1	0.77	28.1
Lysine	14.7	16.8	0.88	17.8	0.83	5.40
Phenylalanine	2.09	2.88	0.69	1.58	0.84	1.10
Threonine	6.48	7.13	0.91	9.04	0.36	1.95
Valine	2.71	3.20	0.91	3.26	0.89	1.42
NEAA						
Alanine	23.5	43.7	0.28	58.2	0.05	14.1
Aspartate	1.98	2.74	0.72	3.17	0.48	1.16
Glutamate	20.2	23.3	0.84	23.2	0.85	6.62
Glutamine	7.06	14.7	0.08	15.5	0.05	3.53
Proline	3.19	5.33	0.38	5.50	0.33	1.75
Serine	8.42	12.8	0.37	7.42	0.93	3.52
Tyrosine	5.39	5.67	0.97	6.25	0.81	1.64

^1^AP = 18% crude protein diet, PR = 9% crude protein diet, AP-R = 18% crude protein diet coupled with rapamycin injections

^2^Data are means and pooled SEM, *n* = 6/treatment

^3^*P-*values correspond to difference between respective treatment and AP by ANOVA with Dunnett *post hoc*

## Discussion

### Adequate dietary protein was unable to sustain lactation performance in mice treated with rapamycin

Overall, mammary mTORC1 signaling was diminished more by AP-R than by PR, but both treatment groups displayed decreased mTORC1 activity on LD13 as evidenced by lower kinase activity (Fig. 2) corresponding with lower pup growth rate and milk production (Fig. 1) as compared to the AP group. Adequate dietary protein is well-established as being critical for sustaining lactation performance across species [22, 36, 37], as the AA from this protein function both as substrate for milk protein synthesis itself and signaling molecules for the pathways governing the synthesis of many milk components [8, 12, 22]. Here, we observed a 15% reduction in pup weight by LD13 in litters nursed by dams on 50% protein-restricted diets, with a similar reduction in pup weight when dams were fed an adequate protein diet and treated with rapamycin. This supports our hypothesis that mTORC1 is required for the regulation of murine lactation by dietary AA, as without its function these dietary AA were unable to sustain lactation performance at AP levels.

In addition to mTORC1 signaling, both glands emptying and suckling stimulus are important regulators of lactation [38]. In cows, incomplete milking is known to decrease milk production through changes in autocrine-paracrine factors [39], in rodents suckling stimulates the arcuate nucleus-Neuropeptide Y system to induce hyperphagia of the dam [40], and in sows prolactin release is triggered by piglets’ manipulation of the glands [41]. It is unsurprising then that dam food consumption was lower in both AP-R and PR treatment groups as lactation progressed. Pups’ failure to thrive initiated by low milk production may have resulted in a negative feedback loop, wherein poor suckling habits contributed to the lack of normal increase in milk production and hyperphagia that was seen in AP dams. However, this possible contributing factor does not diminish the roles of mTORC1 and dietary AA, but rather serves to highlight their critical role in developing and maintaining the multifaceted system of lactation.

### mTORC2 function is not disrupted by intermittent rapamycin treatment

Disruption of the formation of the mTORC2 complex occurs in conjunction with prolonged treatment with rapamycin [42], as the FKBP12-rapamycin complex prevents newly synthesized mTOR from complexing with rictor. As mTORC2 is a distinct kinase complex with functional roles independent of mTORC1, it was necessary to assess whether our rapamycin protocol had off-target affects on mTORC2.

mTORC2 phosphorylates Akt at Ser473 [17], so loss of its formation would have resulted in a decrease in phosphorylation at this site. Intermittent rather than daily treatment with rapamycin is known to prevent the inhibition of mTORC2 formation, as evidenced by sustained Akt (Ser473) phosphorylation [19]. In line with this, in our AP-R dams, Akt(Ser473) phosphorylation was increased by 350% over AP, indicating both successful maintenance of mTORC2 integrity and a loss of negative feedback by S6K1 on PI3K-Akt signaling, as is expected upon pharmacological mTORC1 inhibition [43–45].

### Pup mortality did not play a role in lactation performance

Pup mortality was a minimal issue; two PR dams cannibalized pups (3 on LD2 and 1 on LD7, respectively), but otherwise there were no unintended deaths for the duration of any other lactation (data not shown). While milk production and pup growth are not directly proportional to litter size in mice [46, 47], pup mortality did not impact treatment differences in this study. The low rate of pup mortality is in contrast to Liu et al. [22] who observed complete cannibalization at 9% protein diets when dams nursed litters of 8 pups. These findings suggest that dams can support litter growth at 50% dietary protein restriction when litters are limited to 5 or fewer pups, albeit at a slower growth rate.

### Pup exposure to rapamycin content of milk is unlikely to be an explanatory variable for lactation performance

Because low levels of rapamycin were detected in the livers of pups from AP-R dams, there exists the possibility that the lactation performance of the AP-R group was partly a result of direct pup exposure to rapamycin, rather than solely dam-dependent. Assessing the changes in pup tissue mTORC1 activation would not be a useful metric, as it would not be possible to discern whether changes were due to nutrition or rapamycin exposure when comparing PR and AP-R pups. While AP-R dam livers contained an average 333 ng/g and their pups’ livers only 11 ng/g rapamycin, indicating some limited transfer of rapamycin into milk, it is unclear if the low exposure to rapamycin was sufficient to induce changes to pup growth. However, therapeutic use of rapamycin in human children has been demonstrated to have limited side effects [48], suggesting that even definitively physiologically relevant levels of rapamycin would not result in the decreased growth and milk consumption seen in our study.

Unrelated to our own goals of understanding the molecular mechanisms governing lactation, that rapamycin was present in pup liver tissue indicates not only that the drug is secreted in milk, but that it also can accumulate in offspring consuming that milk. There is a paucity of data on the effects of rapamycin on breastfed infants when the lactating parent is receiving rapamycin treatment [49–51], so although we did not necessarily find evidence for harmful effects of rapamycin exposure on the pups, its accumulation in their livers is worth noting as an unintended side effect.

### Rapamycin may increase levels of some NEAA by downregulating aminotransferase activity

In contrast to Bhasin et al. [52] who found mouse dams fed a 9% CP diet had decreased plasma EAA concentrations, we observed no significant differences for any EAA in refed venous plasma, although all EAA except for Arg and His were numerically decreased. This lack of significant change by diet may be due to timing and sampling location, which can alter blood levels of many compounds in mice [53].

For AP-R, we observed a trend toward increase in refed venous plasma Gln from 661 to 882 μmol/L, and in tissue from 7.06 to 15.5 μmol/L. This is in line with the rapamycin-induced, mTORC1-mediated reduction in cellular Gln uptake and catabolism that occurs as a result of glutamate dehydrogenase (GDH) inhibition by SIRT4, as previously shown *in vitro* [54]. Concordant with reduced GDH activity is a reduction in flux from Glu to α-ketoglutarate (αKG), required for the activity of both aspartate and alanine aminotransferases, which catalyze conversion of Asp and Ala to oxaloacetate and pyruvate, respectively. Limited availability of αKG for aminotransferase activity in AP-R may explain the trend in increase of plasma Asp concentration from 3.72 to 6.27 μmol/L and Ala concentration from 631 to 1.01 × 10^3^ μmol/L in plasma, and tissue Ala rom 23.5 to 58.2 μmol/L.

### Potential for non-mTORC1 regulation of lactation

A factorial design containing a fourth treatment group consisting of dietary protein restriction coupled with rapamycin treatment would have allowed observation of any possible interaction or additive effects. That mammary and liver phosphorylation results (Figs. 2, 3) differed between PR and AP-R even though lactation performance (Fig. 1) was similar indicates that there are factors beyond mTORC1 at play. However, this study was designed solely to test whether inhibiting mTORC1 activity would prevent an adequate dietary supply of AA from supporting lactation, without consideration for other possible regulators, such as the GCN2 pathway [14, 55, 56]. While this is an area that clearly warrants more research, it was beyond the scope of this study, and so treatments were limited to the three described.

## Conclusions

Both protein restriction and systemic inhibition of mTORC1 by rapamycin preferentially affects mammary glands signaling compared to that of the liver in the fed state at LD13, suggesting tissue-specific signaling sensitivity during lactation. More importantly, sufficient supply of dietary AA was unable to maintain lactation performance status in mice with pharmacologically reduced mammary mTORC1 activity, as evidenced by diminished pup growth and milk production, greatly supporting the concept that the substrate role of AA is not the primary factor defining their role in milk synthesis. Rather, the metabolic activity of the mammary glands, regulated through mTORC1 and other pathways, likely defines how and whether those AA will be utilized for lactation.

Systemic effects of rapamycin and other pharmaceutical treatments, as well as potential for off-target effects on pups, indicate a need for a mammary-glands specific genetic approach for the study of mTORC1 function in lactation, which would also aid the understanding of mTORC1-specific function in individual milk component synthesis. However, these results do secure mTORC1’s role as a critical player in the regulation of lactation.

## Data Availability

All data generated or analyzed during this study are included in this published article.

## Abbreviations

4E-BP1: 4E-binding protein 1
αKG: α-Ketoglutarate
AA: Amino acids
Akt: Protein kinase B
AP: Adequate protein diet
AP-R: Adequate protein diet combined with rapamycin treatment
EAA: Essential amino acids
EOD: Every other day
GDH: Glutamate dehydrogenase
IP: Intraperitoneal
LD: Lactation day
mTOR: Mechanistic target of rapamycin
mTORC1: Mechanistic target of rapamycin complex 1
mTORC2: Mechanistic target of rapamycin complex 2
N: Nitrogen
NEAA: Nonessential amino acids
PR: Protein-restricted diet
S6K1: S6 kinase beta-1rp
